# Freeze-Dried Pickering Emulsions with Curcumin: The Role of Stabilizers and Cryoprotectants

**DOI:** 10.1021/acsomega.4c09731

**Published:** 2025-06-27

**Authors:** Lucie Urbánková, Věra Kašpárková, Eliška Dad’ová, Adam Srnec, Petr Humpolíček

**Affiliations:** † Department of Fat, Surfactant and Cosmetics Technology, Faculty of Technology, Tomas Bata University in Zlín, nám. T. G. Masaryka 5555, Zlín 760 01, Czech Republic; ‡ Centre of Polymer Systems, 561989 Tomas Bata University in Zlin, nám. T.G.Masaryka 5555, Zlin 760 01, Czech Republic

## Abstract

This study investigated freeze-dried Pickering emulsions stabilized by a combination of cellulose nanocrystals (CNCs) and sodium caseinate (CAS), with encapsulated curcumin. Our approach focused on the order of CNC/CAS addition and its influence on emulsion properties, along with the effect of three different cryoprotectants (sucrose, d-mannitol, and d-glucose) on the preservation of emulsion droplets. In the study, controlled release of curcumin from freeze-dried emulsions was achieved, attributed to the composition of the stabilizing layer and the cryoprotectant used. The emulsions were partially able to withstand freeze-drying and could be redispersed to samples with droplets bigger than those observed before freeze-drying. The best-preserved droplets came from emulsions stabilized first by CNC particles and then by CAS addition and protected with d-glucose. Transdermal penetration studies revealed that curcumin was mainly present on the skin’s surface and at the *stratum corneum*, with limited penetration into deeper skin layers. Nevertheless, the samples showed outstanding antioxidant activity and no cytotoxicity effects, demonstrating their promising potential to positively influence the healing of the skin.

## Graphical abstract

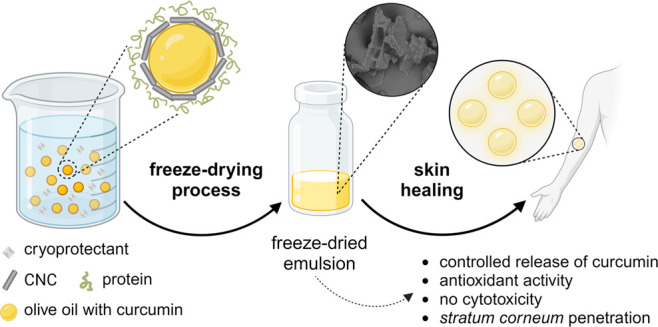

## 1 Introduction

Curcumin is a yellow pigment from *Curcuma longa* and is known for having various beneficial health effects, such as antioxidant, anti-inflammatory, and anticarcinogenic activity.^1^ Due to such biochemical properties, it has also been shown to play a positive role in wound healing. Curcumin, chemically diferuloylmethane, is not a stable substance. It is easily hydrolyzed in alkaline conditions, and UV light causes its degradation. This UV degradation is wavelength-dependent and connected mainly with UV-A and UV-B radiation, at which curcumin undergoes photodegradation, resulting in the breakdown of its molecular structure.^2^ The stability and bioactivity of curcumin are also affected by processing and storage conditions.^1,2^ Furthermore, curcumin is a highly hydrophobic compound; for this reason, its solubility in water is poor. The utilization of curcumin can be improved by developing an efficient delivery system to achieve better solubilization and protecting curcumin from degradation and inactivation. Because of its hydrophobicity, emulsions can be suitable delivery systems for curcumin.^1,3^ Although there are available classic emulsions that show good long-term stability, they can deteriorate with storage time, and the high water content in the formulation might promote microbial growth or cause undesirable reactions in encapsulated substances.^4^ Therefore, the freeze-drying of emulsions can be used to increase their shelf life. Freeze-drying is a dehydration method that uses sublimation to remove water at low temperatures and pressure. Nevertheless, dehydration can lead to disruption of the system being freeze-dried. To protect the emulsion during the freeze-drying process, hydrophilic substances known as cryoprotectants can be added. Cryoprotectants are compounds that prevent stresses from the freezing and drying processes. During freezing, the cryoprotectant can migrate to the concentrated liquid phase, preventing droplet aggregation. Here, saccharides such as glucose (GLU), trehalose, sucrose (SUC), or maltose are widely used.^5,6^ The protective mechanism of saccharides is related to their structures, which contain multiple hydroxyl groups. Saccharides form eutectic mixtures with water, resulting in imperfect ice crystals. Moreover, the hydroxyl groups interact with water, thus increasing the viscosity and decreasing ice crystallization. These mechanisms lead to the inhibition of mechanical stresses exerted on the lyophilized material. The concentration of the cryoprotectant is critical; high concentrations provide better protective activity, but a concentration above the optimal limit can cause system destabilization. The ideal concentration of cryoprotectant is determined by several factors, such as cooling rate and freezing temperature.^6^

During the freezing phase of the freeze-drying process, the oil and water phases start to crystallize. This can cause the collision of oil droplets and their disruption by ice crystals. Droplets covered by a thick stabilizing film have been found to be better protected against penetration by crystals and coalescence. Therefore, Pickering emulsions stabilized by solid particles, which form a dense layer around droplets, can provide better protection against crystal penetration due to the high energy required to remove particles from the o–w interface, thus enhancing resistance to coalescence during freeze-drying and rehydration in comparison with classical emulsions stabilized with surfactants.^5,7^ Moreover, stabilizing particles based on cellulose and proteins are biocompatible and nontoxic, and reduce the risks of irritation or allergic reactions linked to surfactants, making them ideal for pharmaceuticals and cosmetics.^8^ After redispersing, solid particle layers enable controlled release, and emulsions improve the permeability of poorly soluble drugs in delivery applications.^8–10^ Unlike traditional emulsions, which are prone to destabilization from ice crystal formation, Pickering emulsions thus ensure greater stability under freezing conditions.^7,11–15^ The ability of Pickering emulsions to withstand freeze-drying was reported earlier for emulsions stabilized with silica nanoparticles and for redispersible, food-grade and oil-filled powders from octenyl succinic anhydride-modified starch emulsions without the addition of cryoprotectants.^5,16^ Freeze-dried corn oil emulsions stabilized with cellulose nanocrystals (CNCs) combined with cellulose derivatives were also investigated. These emulsions were transformed into solid dry emulsions that suffered from droplet coalescence and could not be redispersed. Adding tannic acid after emulsification, however, allowed for redispersibility by forming a condensed shell around the oil droplets through complexation with cellulose derivatives.^17^

In the current study, we present the preparation and properties of freeze-dried Pickering emulsions with incorporated curcumin. The emulsion formulation was based on earlier studies in which combinations of CNC and sodium caseinate (CAS) were employed to stabilize the emulsions.^18,19^ When compared to synthetic or nonbiodegradable stabilizers, CNC and CAS distinguish themselves by their natural origin, biocompatibility, biodegradability, and positive environmental impact. These characteristics make CNC and CAS appealing choices for emulsion stabilization. At the beginning of our research, we hypothesized whether CNC/CAS/curcumin Pickering emulsions could be freeze-dried with the aid of saccharide-based cryoprotectants and how the cryoprotectant type would affect the decisive characteristics of the emulsions (both prior to and after freeze-drying), including droplet size and distribution, encapsulation efficacy, reconstitution, and the curcumin release profile. The produced emulsions were examined for their antioxidant activity (AA) as well as their biological performance in terms of transdermal penetration and cytotoxicity, which are essential for wound healing applications.

## 2 Results and Discussion

Prior to emulsification, the interfacial tensions of the CNC dispersion, CAS solution, and water were measured at the interfaces with olive oil (OO) or olive oil containing curcumin (OO-Cur). Figure 1 shows that there were minor differences in the interfacial behavior of systems containing virgin OO or OO-Cur, which, however, were not significant (*p* ≥ 0.5). The interfacial tension in the presence of aqueous CNC dispersions ranged from 19.6 ± 0.9 to 21.2 ± 0.6 mN/m. In systems with aqueous CAS, the values were lower both in the presence of OO and OO-Cur, being 12.1 ± 0.5 and 11.6 ± 0.3 mN/m, respectively. This was expected due to the surface activity of CAS, as documented in Pind’áková et al. 2019.^18^

**Figure 1:**
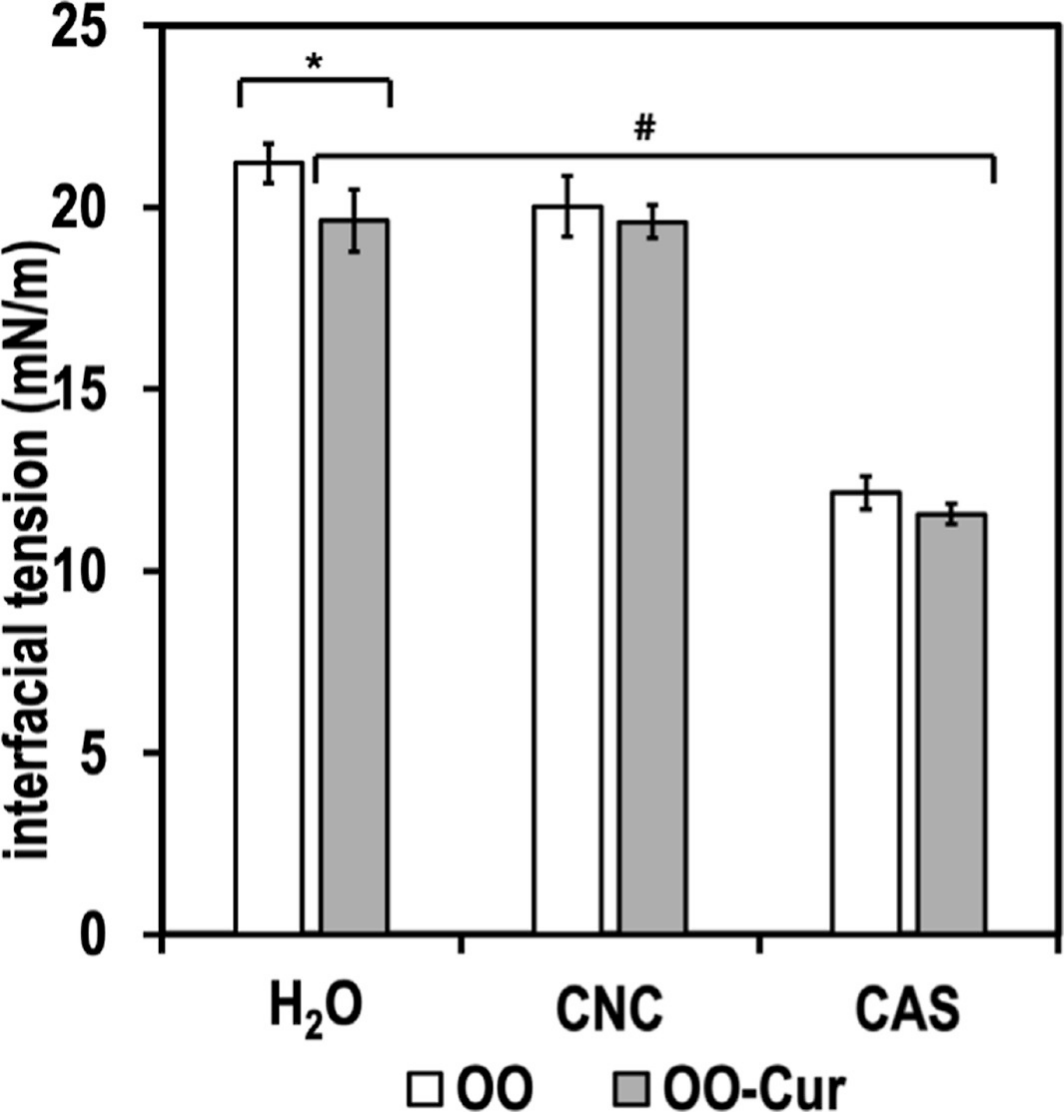
Interfacial tensions of CNC dispersion, CAS solution, and water interface with OO and OO-Cur. In figure * express the statistical difference between samples with the same aqueous phase and different oils (*p* ≥ 0.05). # Express the statistical difference between the different aqueous phases (*p* ≥ 0.05).

### 2.1 Emulsions

In order to prepare freeze-dried emulsions that would serve as curcumin carriers, oil-in-water emulsions stabilized by combinations of CNC and CAS added in different orders (routes E1, E2, and E3) were formulated. The emulsion oil phase contained curcumin dissolved in OO, and its use was motivated by OOs ability to improve cutaneous wound healing, reduce oxidative damage and inflammation, promote the repair of epithelial tissue, and accelerate the overall healing process.^20–22^ These properties result from the presence of phenolic compounds in the oil.^20–22^ To protect the emulsion during the freeze-drying process, various saccharide-based cryoprotectants, namely, SUC, mannitol (MAN), and GLU, were used.

#### 2.1.1 Droplet Size and Distribution

The sizes of the emulsion droplets varied depending on the route of emulsion preparation and composition of the oil phase, which contained OO or OO-Cur. However, irrespective of the oil’s phase, E2 emulsions stabilized primarily by CAS and then CNC exhibited the largest droplets in comparison to the E1 (CAS + CNC simultaneously) and E3 (first CNC followed by CAS) emulsions. Moreover, the final E3 emulsion had the smallest and most stable droplets. Therefore, the synergistic effect of Pickering stabilization with CNC in the first step, followed by the addition of surface-active CAS in the second step, provided the best stabilization for the studied samples. The high emulsifying capacity of route E3 was apparent from the absence of free, nonencapsulated oil observed already in the primary emulsion stabilized solely by CNC containing relatively large droplets. The subsequent addition of CAS resulted in a significant reduction in droplet size, which was due to the strong CNC-CAS synergy. Given that CAS has a higher interfacial activity than CNC, its addition likely facilitated an increase in the droplet curvature by reducing the interfacial tension. A possible mechanism for this size reduction could be the partial displacement of CNC by the CAS at the o–w interface. However, this is unlikely, as CNC particles have a high desorption energy once they are adsorbed at the o–w interface. Therefore, the improved stabilization of emulsions prepared by route E3 is most likely due to a combination of Pickering stabilization (CNC) and reduction of interfacial tension (CAS). This trend observed for emulsions containing OO and OO-Cur is consistent with previous findings for emulsions encapsulating hexadecane oil.^18^

However, under more detailed inspection, it was observed that curcumin dissolved in the oil phase influenced the size and distribution of emulsion droplets prepared using all three routes. Emulsions without curcumin in the oil phase exhibited somewhat larger droplet sizes, ranging from 3.1 to 8.0 μm, than curcumin emulsions (OO-Cur), with droplet sizes ranging from 2.4 to 7.5 μm (Figure 2a). Although the size differences were relatively small, this trend was observed for all emulsions. Curcumin is most likely not only a bioactive substance but also plays an active role in the stabilization of emulsions. The ability of curcumin to contribute to the stabilization of oil-in-water emulsions was reported earlier, and it was revealed that even curcumin particles alone facilitate the preparation of emulsions by means of Pickering stabilization.^23^ Nevertheless, curcumin dissolved in the oil phase can also have a stabilizing effect—for example, by means of promoting stabilizing agents to form a larger total area of interfacial film.^24^

**Figure 2:**
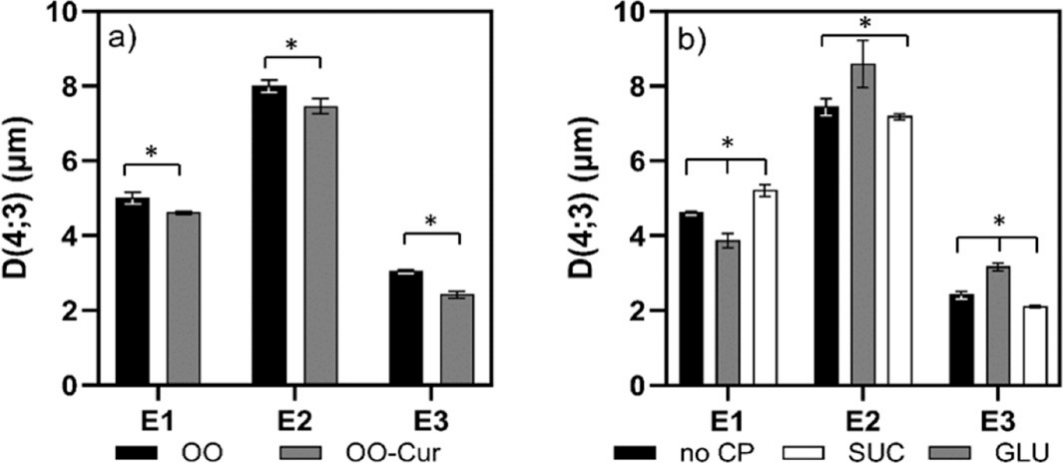
Effect of (a) curcumin and (b) cryoprotectants on the size of emulsion droplets prior to freeze-drying. OO denotes emulsion with olive oil and OO-Cur denotes OO with added curcumin. Emulsions were prepared by procedure E1 (addition of CAS + CNC simultaneously), E2 (addition of CAS, then CNC), and E3 (addition of CNC, then CAS), without cryoprotectants (no CP) and with sucrose (SUC) and glucose (GLU).

The presence of cryoprotectants is another parameter that affected the droplet size and stability of emulsions. Because the efficacy of sugar-based cryoprotectants with respect to droplet stabilization under freeze-drying depends on their type and concentration, MAN, GLU, and SUC were chosen, and their amount was fixed at 10 wt % of the total emulsion volume, according to an earlier published study.^25^ The droplet sizes of emulsions with MAN could not be determined by diffraction measurements, as this cryoprotectant caused droplet aggregation, which was not observed in emulsions with GLU and SUC. Similarly, MAN caused the aggregation of poly­(epsilon-caprolactone)/poly­(vinyl alcohol) nanocapsules during freeze-drying.^25^ The reason for this is that MAN crystallizes and forms eutectics with ice,^25^ resulting in phase separation in the cryo-concentrated part of the frozen emulsion with no possibility for a stabilizing contact with nanocapsules.

In the presence of cryoprotectants, the effect of the preparation route on droplet size corresponded with the effect observed in emulsions in which cryoprotectant was absent. Figure 2b shows a comparison of the droplet sizes of emulsions with GLU and SUC prepared with and without cryoprotectants and illustrates that the addition of 10% SUC to the emulsions had no effect on the droplets. This is also clear from Figure 3a, which shows the distribution curves. Given that the distributions of the emulsions are almost identical, the addition of SUC did not influence the size of the droplets or likely the stabilization of the emulsions. This observation is also supported by microscopic images of the emulsions (Figure 4). More detailed examination of the microscopic figures reveals that, in all cases, the emulsions contained three populations of particles. The distribution curves for emulsions with GLU followed a multimodal trend similar to that for samples with SUC. The effect of added GLU on droplet diameter *D*(4; 3) was more significant, with E1 emulsion droplets with GLU being smaller than those without. This difference is shown in Figure 4, where the microstructure of emulsions is displayed. With the addition of GLU, the *D*(4; 3) of the E2 and E3 emulsions was slightly increased. In the case of E2 samples, this could be caused by the flocculation of droplets, as shown in Figure 4. Emulsion E3-GLU was homogeneous and free of flocs. MAN emulsions were examined only according to their microstructure visualized by microscopy. Compared with emulsions without MAN, the E1-MAN emulsions contained larger droplets. The E2-MAN emulsion was not homogeneous and contained flocculated droplets. The E3-MAN emulsion showed higher homogeneity than the E1-MAN emulsion, and both samples exhibited an emulsion network that was relatively dense and cohesive, which may have complicated laser diffraction measurements.

**Figure 3:**
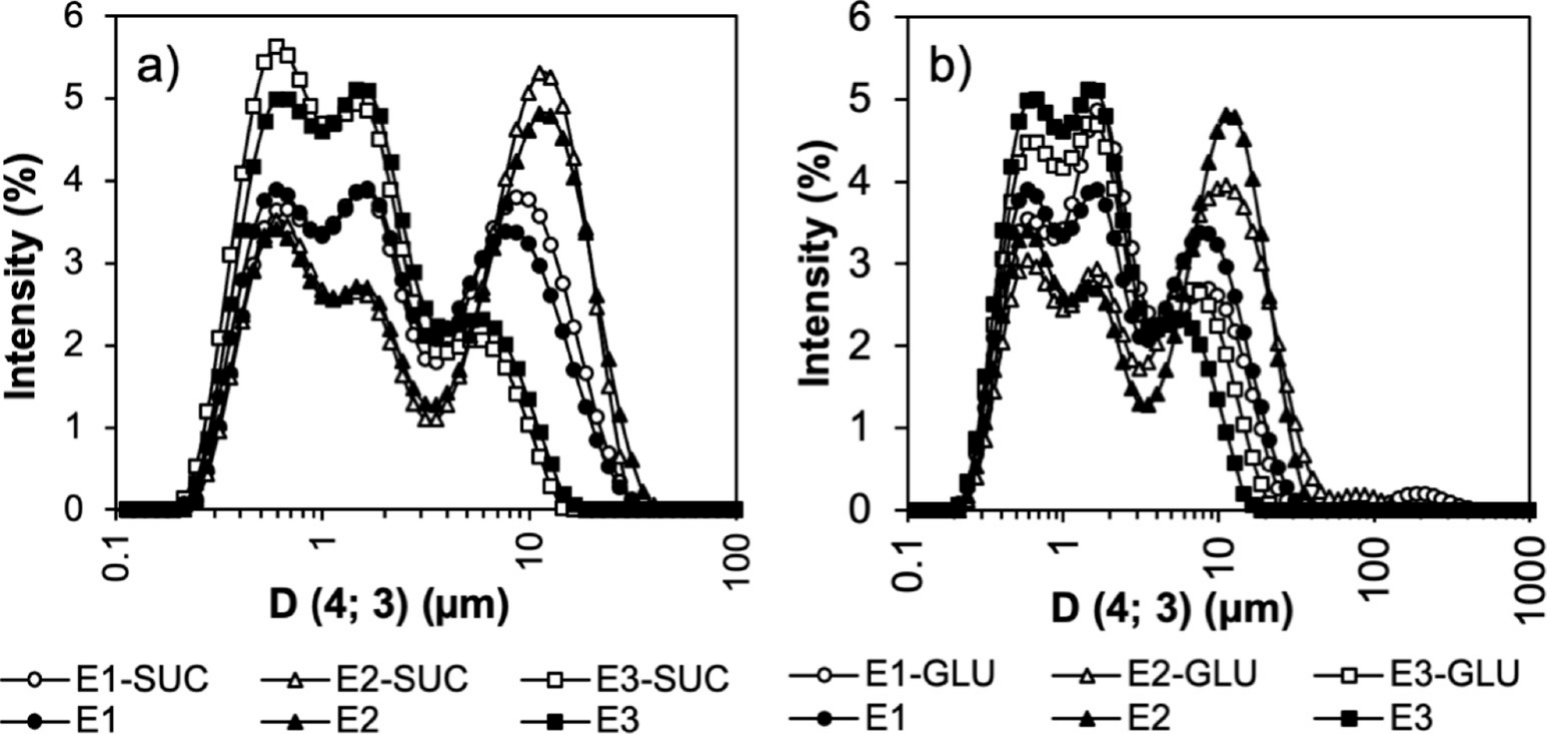
Distribution of droplets in emulsions with OO-Cur prepared via the E1, E2, and E3 routes (a) with SUC and (b) with GLU as cryoprotectants, compared with the emulsions without cryoprotectant.

**Figure 4:**
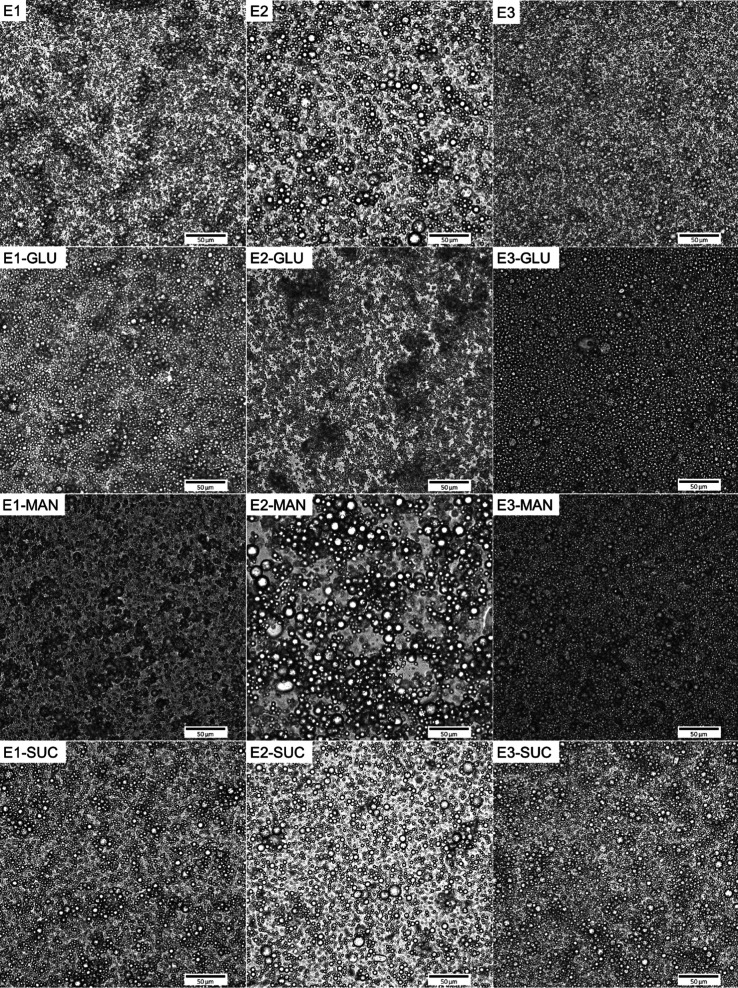
Microstructure of OO-Cur emulsion droplets prepared by the E1, E2, and E3 routes without cryoprotectant (first row), and with E-GLU, E-MAN, and E-SUC, visualized by microscopy, scale bar is 50 μm.

### 2.2 Freeze-Dried Emulsion

The freeze-drying of an emulsion is a way of extending its shelf life, improving its utilization, and facilitating transportation. This advantageous process is associated with the removal of water, which accelerates the degradation of stabilizing polymers (here CAS), oils, and curcumin, which is prone to degradation, particularly in an alkaline aqueous environment.^5,26^ After successful freeze-drying, the emulsion cake should have a well-formed shape without sunken or bulging centers, and the cake should touch the walls of the vial.^27^ The freeze-dried emulsions without cryoprotectant prepared as a reference in this study did not meet these requirements. The quality of cakes was poor, with destabilized structures and visible oil leaks (Figure 5a,b). Thus, our observation agrees with the study in which emulsions stabilized by CNCs alone displayed extensive emulsion breaking and oil leakage after freeze-drying.^17^ In contrast, visual observation of the studied samples with cryoprotectants demonstrated that the structure of all freeze-dried cakes was compact, with no collapses or separate phases. The cakes were homogeneous in color, indicating good distribution of curcumin. As an example, Figure 5c shows the FDE-MAN cake, which serves as a representative illustration of the appearance of all freeze-dried emulsions with cryoprotectants. Detailed observation revealed that the freeze-dried cake with SUC had a fine structure with a waxy-like texture. MAN-containing samples had the texture of a fine fluffy powder, which easily deteriorated in contact with air moisture, thus losing its original structure when manipulated. Freeze-dried emulsions with GLU had a cotton candy appearance and transformed into oily particles under manipulation. The differences in the texture of the freeze-dried emulsions arose primarily because of the different types of cryoprotectant used. However, the freezing procedure is another parameter that cannot be ignored because it also influences the properties of the lyophilizate and its crystal structure.^28^ The crucial parameters are the freezing temperature, cooling rate, cryoprotectant concentration, and sublimation time. In a published study, a lyophilizate (an OO emulsion with xanthan gum) with MAN as the cryoprotectant resulted in the best physical properties, structure, and redispersion in comparison with the same lyophilizate containing erythritol and lactose as cryoprotectants.^29^ Water-soluble polymers such as methyl cellulose or hydroxyethyl cellulose can also act as cryoprotectants when added to CNC-stabilized Pickering emulsions.^17^ Additionally, freeze-drying was used to prepare redispersible, oil-filled powders from Pickering emulsions stabilized with OSA-modified starch without the addition of cryoprotectants.^5^

**Figure 5:**
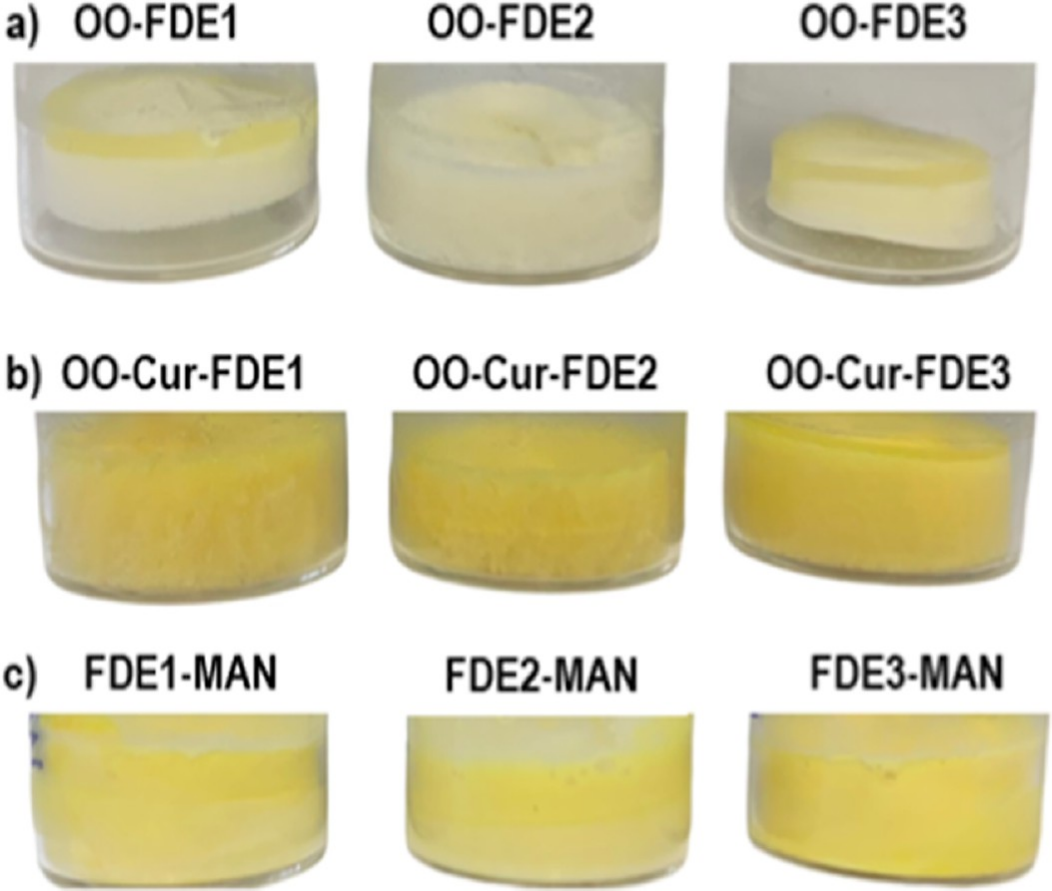
Comparison of freeze-dried emulsion cakes (a) OO emulsions without cryoprotectant, (b) OO-containing curcumin (OO-Cur) emulsions without cryoprotectant, and (c) OO-containing curcumin (OO-Cur) emulsions with the cryoprotectant MAN, each prepared with routes E1, E2, and E3.

#### 2.2.1 Microstructure of Freeze-Dried Emulsion

The microstructures of the freeze-dried emulsions were visualized by scanning electron microscope (SEM) (Figure 6) and complied with the visual assessment. MAN-containing samples had a more crystalline and powdery structure than samples with GLU and SUC, making them easier to differentiate at first sight. The freeze-dried samples with GLU and SUC, in contrast, were compact. The structure of the freeze-dried cake reflected the structure and shape of the cryoprotectant’s crystals. In some cases, emulsion droplets surrounded by solid particles could be observed in the cake (see insert picture in FDE1-GLU). This observation shows that the emulsion droplets survived the freeze-drying process and remained preserved in the cryoprotectant matrix.

**Figure 6:**
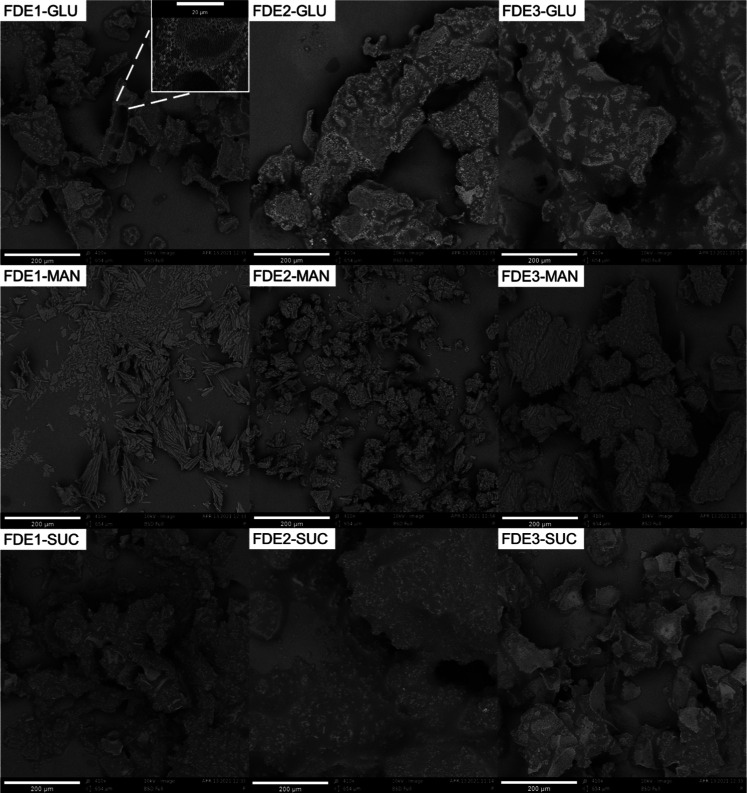
Microstructure of freeze-dried emulsions (FDE) observed by SEM. FDE1, FDE2, and FDE3 correspond to the different routes of preparation; SUC, GLU, and MAN denote the cryoprotectants used. Scale bar is 200 and 50 μm for insert in FDE1-GLU.

#### 2.2.2 Redispersion of Freeze-Dried Samples

Testing the redispersion of the freeze-dried emulsions in water provided evidence that the emulsion droplets could maintain their integrity during freeze-drying. However, after the addition of water to FDE in the amount corresponding to the original o/w ratio of 20/80, the redispersion was difficult to accomplish; the reconstituted emulsions were flocculated and nonhomogeneous. Nevertheless, adding an amount of water to the FDE samples in a ratio 50/50 (w/w) gave rise to samples, which were homogeneous and retained the properties of the concentrated emulsions. Upon additional dilution with water to o/w 20/80, the emulsions slightly flocculated but not as much as when water was added directly to the 20/80 ratio. Microscopy pictures of reconstituted emulsions with GLU and SUC are depicted in Figure 7 and show fully preserved droplets of various sizes together with agglomerated ones. An oil leak is visible only in the emulsion prepared with route 2 and SUC (FDE2-SUC). Comparison with microscopy pictures of the corresponding emulsions captured before freeze-drying (Figure 4) demonstrates an increase in the droplet size for most of the samples after being freeze-dried. As measured by light diffraction, the size of emulsion droplets before freeze-drying with SUC and GLU ranged from 0.2 to 20 μm (Figure 3), with *D*(4; 3) not exceeding 8 μm. The emulsions containing MAN were not measured due to sample aggregation caused by its presence. After freeze-drying, image analysis revealed an increase in the droplet diameter, which was mostly controlled by the cryoprotectant type used. The FDE1-GLU sample was well-preserved and contained predominantly droplets with a size of about 5 μm with a uniform distribution; however, larger droplets were also observed. Redispersed FDE2-GLU and FDE3-GLU emulsions contained in addition to 5 μm droplets also bigger ones (60–70 μm) together with sporadically observed ∼200 μm droplets. Droplets of emulsions redispersed from FDE-SUC were smaller (∼2–3 μm) and were accompanied by bigger ones with sizes ranging from 70–300 μm. Freeze-dried samples with MAN also showed the poorest performance after freeze-drying. As regards stability after redispersion, the emulsions freeze-dried with SUC and GLU did not show creaming, while for MAN emulsions, creaming was typical irrespective of the route of emulsion preparation. From the test, it was obvious that the used cryoprotectants were able to protect emulsions under freeze-drying only to a certain extent because the droplet sizes in redispersed emulsions differed from the original ones. Therefore, we decided not to monitor the long-term stability of these samples. A review of relevant scientific studies shows that freeze-drying Pickering emulsions is not a straightforward process. Here, silica nanoparticles and starch granules are mostly reported as Pickering stabilizers used under freeze-drying. The successful redispersion of freeze-dried emulsions stabilized with CNC was reported; however, the study used CNCs preadsorbed with methylcellulose or hydroxyethyl cellulose in combination with tannic acid to enhance the preservation of droplets under the freeze-drying process.^17^

**Figure 7:**
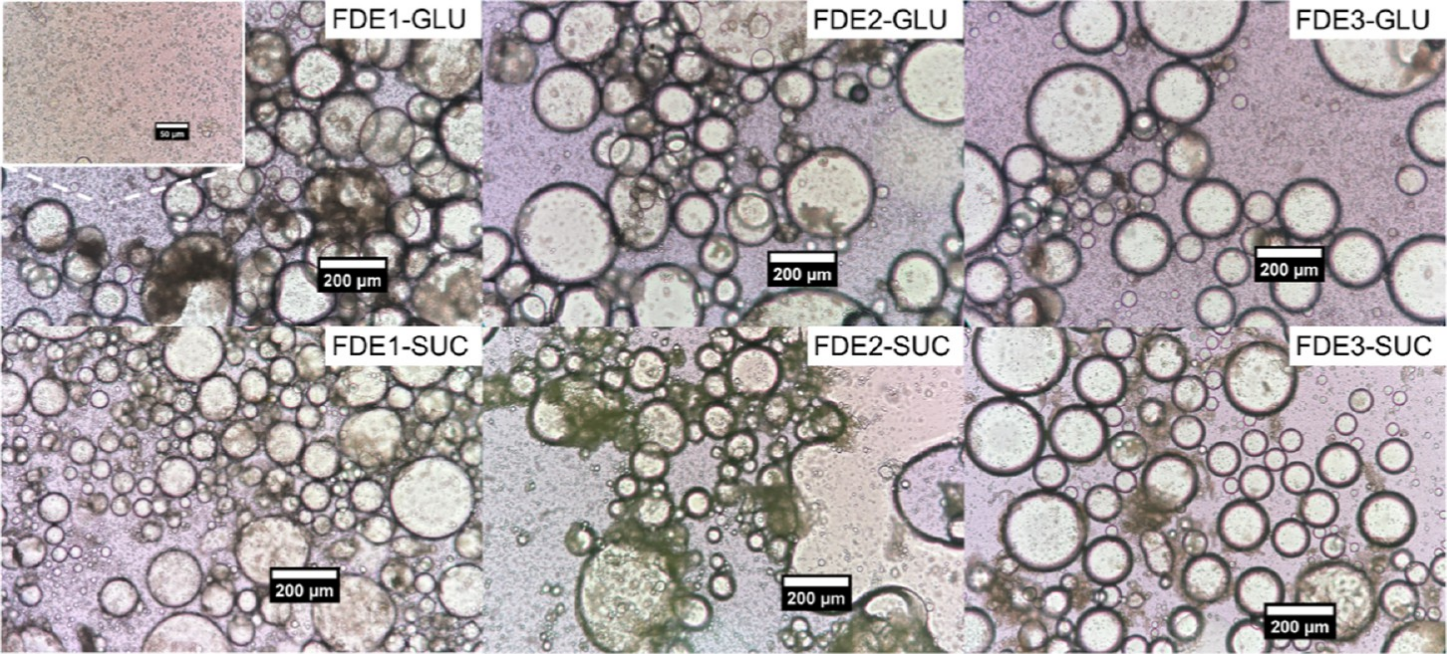
Microstructure of redispersed FDE with GLU (first row) and SUC (second row) as cryoprotectants prepared with different routes of emulsification. Scale bar is 200 mm.

#### 2.2.3 Release of Curcumin from Freeze-Dried Emulsions

Due to the low solubility of curcumin in water (<0.6 μg/mL),^30^ and in order to better simulate physiological conditions, curcumin release from freeze-dried samples was determined using phosphate-buffered saline containing 0.5 wt % Tween 80. The curcumin release profiles vs time and total amounts of curcumin released are depicted in Figure 8. In the first few hours, the data show an initial burst release, followed by a mild release of the encapsulated substance. The amount of curcumin released correlated with the type of cryoprotectant used. Within the first 3 h, 2–4% curcumin was released from freeze-dried GLU emulsions, 2–3% from MAN-containing sample, and those with SUC had the least tendency to release curcumin (1.5–2.5%). After 24 h, the ability of the studied freeze-dried emulsions to release curcumin remained in the same order as in the first hours of the test and was as follows: 5.5–6% for FDE-GLU, 4–5% for FDE-MAN, and 3–4% for FDE-SUC. The curcumin release then gradually and slightly increased and, after 72 h, the maximum release was observed for FDE-GLU (8–13%), followed by 7–9% for FDE-MAN and 5–7% for FDE-SUC. Curcumin release from FDE was highest for samples formulated with GLU (13%), and a greater amount of released curcumin was observed for samples prepared using routes E1 and E2 than for samples prepared using route E3 (Figure 8d). Thus, it can be seen that the formulation/composition of the freeze-dried emulsion influenced the release of curcumin from the matrix and that the choice of cryoprotectant and the route of emulsion preparation (the composition of the stabilizing layer of droplets) can be effectively combined to control curcumin release.

**Figure 8:**
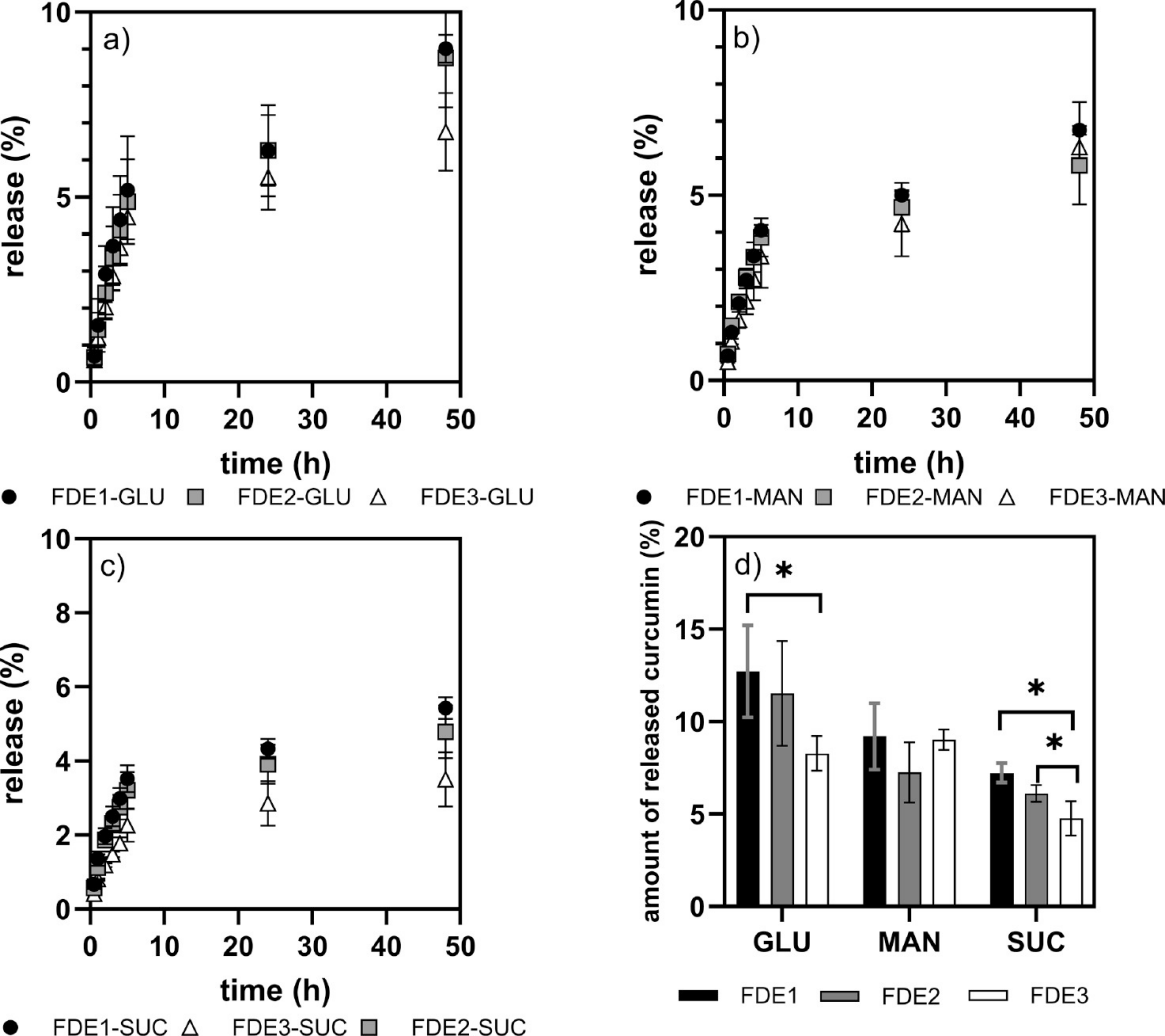
Course of curcumin release from FDE during a time interval of 50 h. Emulsions freeze-dried with (a) GLU, (b) MAN, (c) SUC, and (d) cumulative curcumin release after 72 h.

Previously published studies on curcumin release from Pickering emulsions stabilized with chitosan-tripolyphosphate nanoparticles found that 37 and 42% of curcumin was released after 24 h into a buffer medium with pH 7.4.^31^ In another study, solid lipid nanoparticles and nanostructured lipid carriers released 30–50% of the encapsulated curcumin after 24 h.^32^ It is obvious that the amount of curcumin released after 24 h in the current study is lower than that reported in the above-mentioned studies. Nevertheless, even such a comparatively low curcumin release can contribute positively to skin healing. The reduced release observed in our study is explained by the fact that freeze-dried emulsions are not the same as Pickering emulsions or solid lipid nanoparticles as delivery systems. Temperature, pH, and experimental settings, as well as sample diffusion and degradation, are all important factors, which must also be considered in these in vitro release studies.^33^ In this regard, a well-chosen delivery system can prolong the drug release time while also protecting the active substance, thus improving its efficacy.^34^ Furthermore, as curcumin is a polyphenolic compound, its topical application at high concentrations can occasionally lead to a toxic response. Therefore, a slower and gradual release may be advantageous in this respect, and selecting an optimal delivery system is important when using curcumin for skin healing. The main goal of an appropriately chosen carrier system is to improve curcumin solubilization, protect it from inactivation by hydrolysis, and control the release of the drug in solubilized form.^1^ In our opinion, freeze-dried emulsions are suitable delivery systems for slow curcumin release.

#### 2.2.4 Radical Scavenging Activity

Curcumin is known for its AA. To confirm this for the investigated freeze-dried emulsions, their free radical scavenging activity was determined using 2,2-diphenyl-1-picrylhydrazyl (DPPH) radicals. The scavenging activity was measured at predetermined intervals over 60 min, beginning with the mixing of DPPH with the freeze-dried emulsions. The results showed that the AAs of samples with different cryoprotectants and different preparation routes ranged from 58 to 75%, and that the curcumin emulsions exhibited radical scavenging activity already during the first minutes of contact with radicals, when AA increased rapidly (Figure 9). Although there was no clear correlation between AA and the route of emulsion preparation, MAN-containing FDE showed a low repeatability of AA values, which can be attributed to the poor quality of the FDE-MAN cake after manipulation, as discussed in Section 2.2. In order to validate the contribution of OO to the AA of our emulsions, the test was conducted with 0.1 mM DPPH ethanolic solution and OO after being mixed 1:1; here, OO demonstrated scavenging activity of 87%. However, no scavenging activity was seen when OO was tested in a quantity equivalent to the oil concentration in 50 mg of a freeze-dried sample (the amount used to test AA on final freeze-dried formulations). Hence, the scavenging activity of FDE emulsions is due solely to the presence of curcumin.

**Figure 9:**
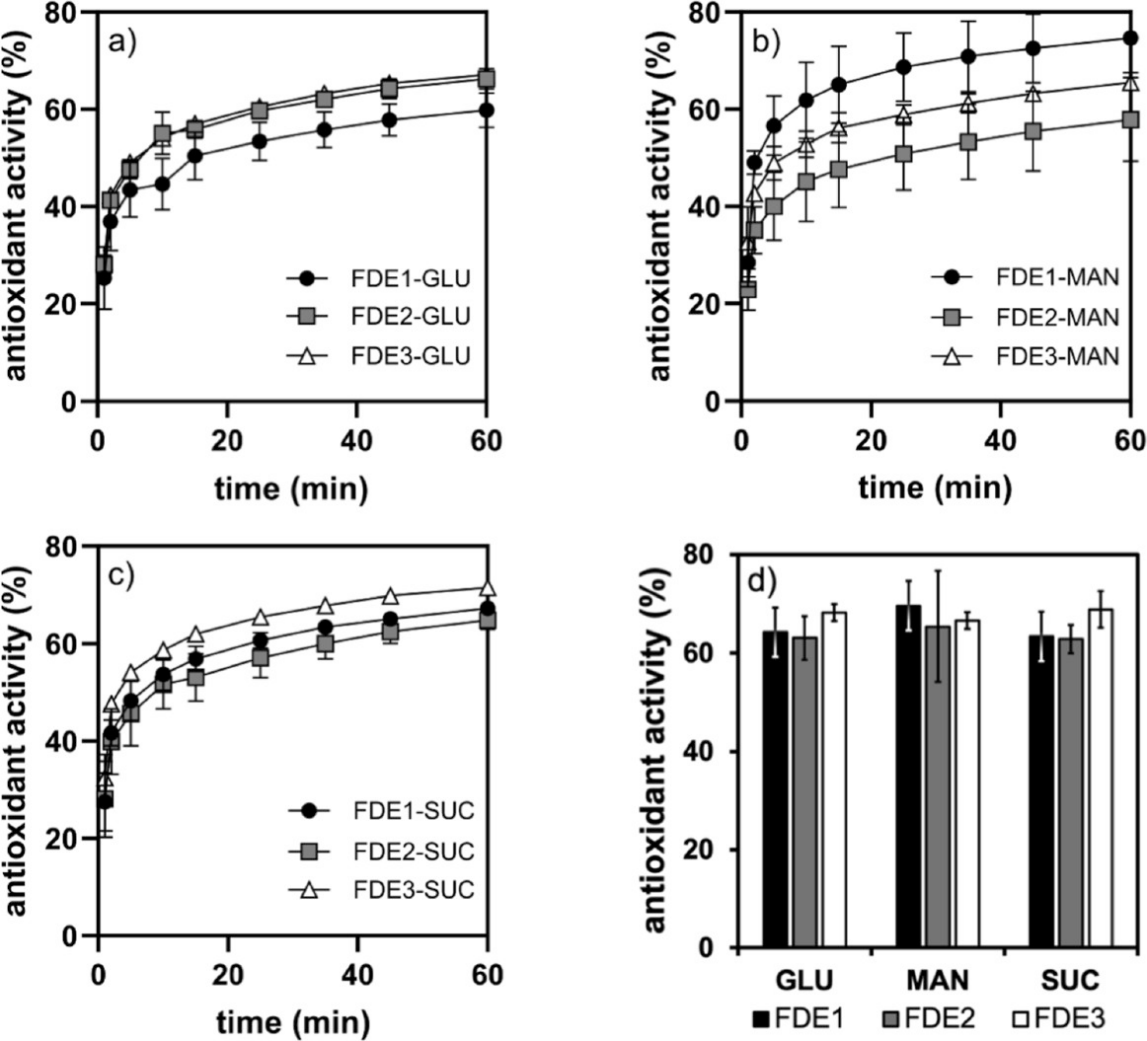
AA of emulsions freeze-dried with cryoprotectants (a) GLU, (b) MAN, (c) SUC, and (d) AA after 60 min.

Previously, the scavenging activity of curcumin dissolved in triacylglycerol-based oils encapsulated in two different carrier systems, Pickering emulsions (stabilized with chitosan-tripolyphosphate nanoparticles) and nanoemulsions (stabilized with Span 80/Tween 80), was reported.^31^ In this work, the AA ranged from 29.8 to 49.6% after 45 min, depending on the type of emulsion and oil. Nevertheless, the ability of curcumin to scavenge radicals is dependent on its concentration and the type of carrier system used.^35^

### 2.3 Biological Performance

Curcumin, due to its antioxidant and anti-inflammatory properties, can enhance wound healing when given topically.^1^ Therefore, the transdermal penetration of curcumin from FDE was investigated in an in vitro porcine ear model; in addition, emulsion cytotoxicity was assessed.

#### 2.3.1 Transdermal Penetration

Curcumin skin penetration was investigated with freeze-dried emulsions made via route E3, which demonstrated the best retained droplets. Before being applied to the skin, freeze-dried samples were mixed with water in a 1:1 ratio, which may mimic the conditions under which moist wound healing takes place. The amount of curcumin in individual skin layers was determined by using UV/vis spectrophotometry after extracting curcumin in methanol (Figure 10). The curcumin encapsulated in FDE showed relatively low penetration, with the majority of the applied amount remaining on the skin surface. The main differences in penetration pattern were observed among formulations containing various cryoprotectants. Emulsions freeze-dried with MAN demonstrated the poorest penetration. Curcumin remained on the skin’s surface, with only a trace detected in the first layers of the *stratum corneum* (strips 1 and 2, 1.3%), and was absent in lower skin layers and receptor fluid. Curcumin from FDE with GLU and SUC penetrated slightly better but was not detected in the receptor fluid or *dermis* in the case of FDE with GLU. Curcumin penetration into the *epidermis* was 0.24 and 0.10% for FDE-GLU and FDE-SUC, respectively. For FDE with SUC and GLU, 8.1 and 9.0% of the applied curcumin amount were found in the *stratum corneum* (strips 1–6), respectively. Interestingly, a previously published study^36^ found that the initial concentration of curcumin is an important determinant of the depth of its penetration into the skin (Fick’s law). Curcumin was found to penetrate to the *stratum corneum* at lower concentrations, to the *epidermis* at ≥0.15%, and to the *dermis* at concentrations higher than 0.25%. In our study, the total curcumin content in applied samples was 0.062%, implying that curcumin penetration from the studied FDE to the dermis and receptor fluid was unlikely.

**Figure 10:**
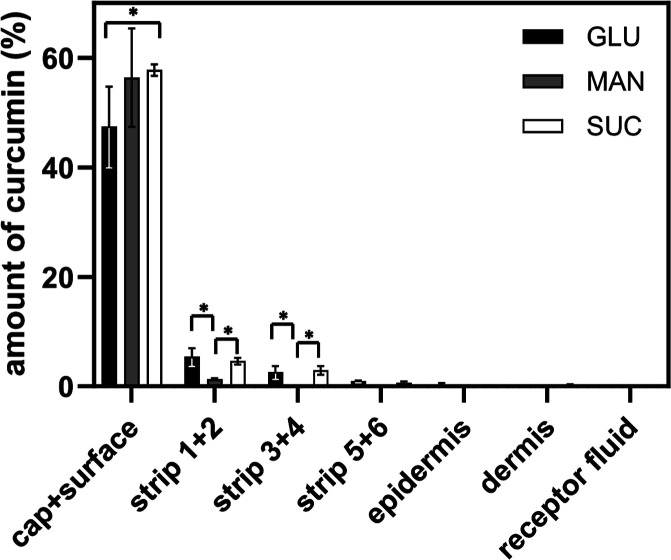
Transdermal penetration of curcumin from emulsions freeze-dried with GLU, SUC, and MAN.

#### 2.3.2 Cytotoxicity

Cytotoxicity is a key factor in determining whether a material is suitable for applications in contact with tissues and cells. Moreover, in terms of dermal application, it might serve as an indirect measure of cutaneous irritation. The in vitro cytotoxicity of curcumin emulsions toward NIH/3T3 mouse embryonic fibroblast cells was determined, and the data were evaluated in accordance with ISO 10993-5 requirements, which define cytotoxicity as cell viability lower than 0.7 in comparison to the reference. The MTT results given in Figure 11 show that cytotoxicity correlates with both the route of emulsion preparation and the type of cryoprotectant used. The samples with SUC, regardless of the preparation route, displayed the lowest cytotoxicity and were cytotoxic first at 50% dilution, corresponding to 38 μg of curcumin. In contrast, emulsions with GLU showed the highest cytotoxicity, with a threshold that varied depending on the route of emulsion preparation. Emulsions prepared with route E2 were cytotoxic at 5% dilution (3.8 μg curcumin), while route E3 produced emulsions with a cytotoxicity threshold of 19 μg curcumin (50% dilution). In general, regardless of the cryoprotectant used, emulsions produced using route E3 exhibited the lowest cytotoxicity. The results thus show that cytotoxicity correlates with the amounts of curcumin released from emulsions prepared with different cryoprotectants, which decrease in the order GLU → MAN → SUC-protected emulsions, and that preparation route E3 (CNC first followed by CAS) provides the best stabilizing layer for curcumin encapsulation.

**Figure 11:**
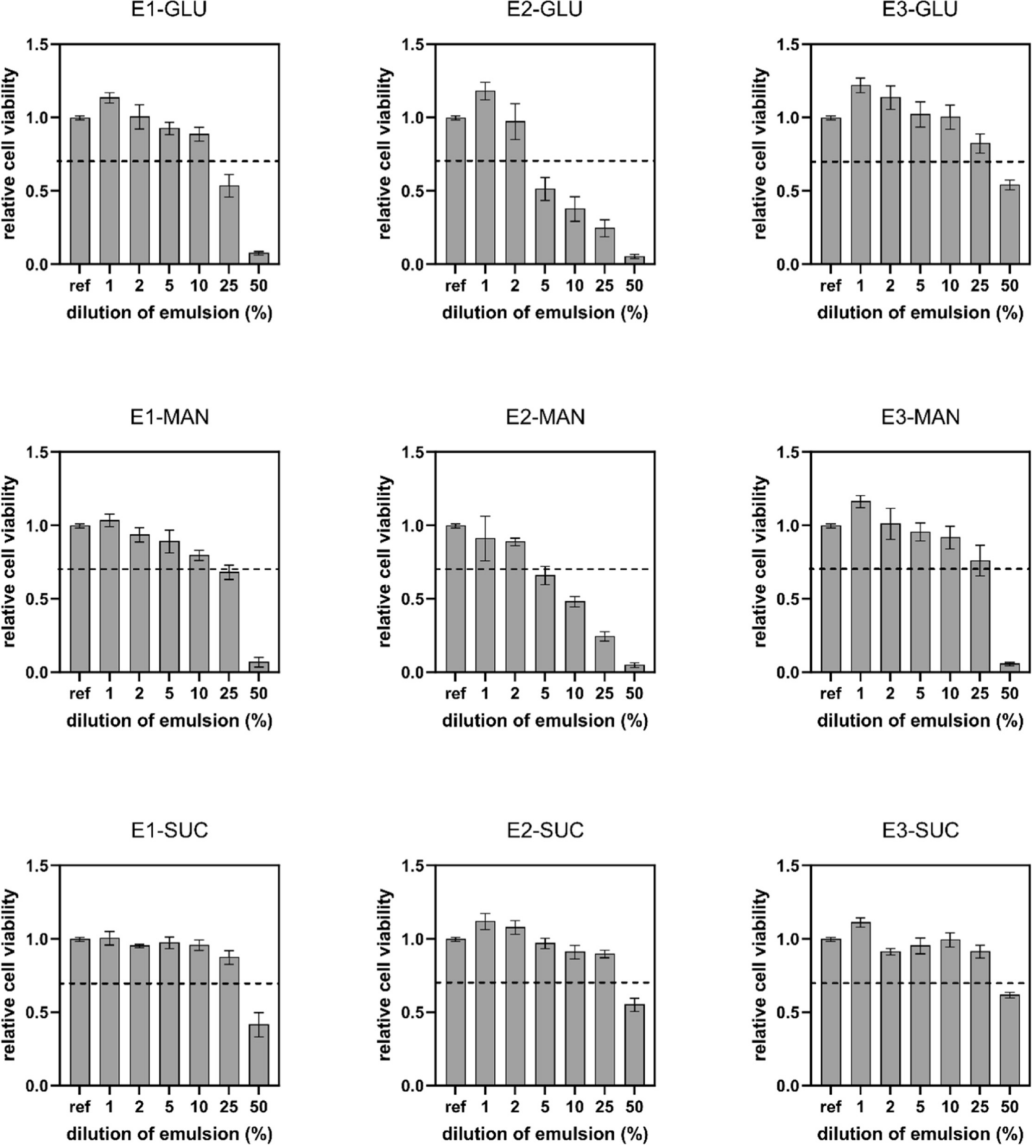
Cytotoxicity of emulsions determined using MTT assay. The dashed lines highlight the limits of cell viability according to EN ISO 10993-5: viability >0.7 corresponds to the absence of cytotoxicity.

## 3 Conclusion

Pickering stabilization with CNC combined with surface active CAS was used to create emulsions containing curcumin dissolved in OO. The effect of the oil phase and order of stabilizer addition (CNC and CAS) on emulsion properties was investigated along with the properties of freeze-dried emulsions. Here, the effect of three different cryoprotectants (SUC, d-MAN, and d-GLU) on the preservation of emulsion droplets was investigated together with AA, the transdermal penetration of curcumin, and the cytotoxicity of the emulsions. The study revealed that emulsions prepared with CAS and CNC stabilizers were able to withstand freeze-drying only to some extent and could be redispersed to samples with droplets bigger than those observed before freeze-drying. The best-preserved droplets came from emulsions stabilized first by CNC particles followed by CAS addition and protected with d-GLU. The tests demonstrated that curcumin release from freeze-dried emulsions can be controlled by varying the composition of the stabilizing layer, that is, by changing the order of CAS and CNC addition. Moreover, curcumin can be slowly released at the application site as a result of the gradual erosion of the freeze-dried cake, which is controlled by the cryoprotectant type. Transdermal penetration studies revealed that curcumin showed minimal penetration into deeper skin layers and was mostly found at the *stratum corneum* and on the skin surface. These properties, combined with the samples’ documented AA and absence of cytotoxicity, indicate the promising potential of such emulsions to enhance the healing of skin.

## 4 Materials and Methods

### 4.1 Materials

CNC powder with the commercial name CelluForce NCC obtained via sulfuric acid hydrolysis was purchased from CelluForce (Canada). The particles were of a typical rod-like shape of length 80–220 nm and diameter 4 nm, as determined by AFM. Zeta potential measurements as a function of pH demonstrated potential values of −36 to −66 mV in the pH range 4–10. Detailed information on the properties of the CNC used is given in ref 18. Casein sodium salt from bovine milk (CAS) and curcumin from *C. longa* (≥65%) were purchased from Sigma-Aldrich (Germany). Extra virgin OO was purchased from a local store. Sodium chloride (NaCl), Tween 80, Brij 98, gentamicin sulfate, and 2,2-diphenyl-1-picrylhydrazyl (DPPH) were obtained from Sigma-Aldrich and Merck (Germany) in analytical grade. SUC, d-MAN, and d-GLU were purchased from Chemapol and Penta (Czech Republic). Dulbecco’s phosphate buffered saline (PBS) was obtained from Biosera (France), and Spectra/Por standard grade RC dialysis membrane (3.5 kDa) was from Repligen (USA).

The ATCC CRL-1658 NIH/3T3 mouse embryonic fibroblast cell line, USA, was used for cytotoxicity testing. ATCC–formulated Dulbecco’s modified Eagle’s medium (PAA Laboratories GmbH, Austria), containing 10% calf serum (BioSera, France) and 100 U·mL^–1^ penicillin/streptomycin (GE Healthcare HyClone, United Kingdom), was used as the culture medium. All studies were conducted using Milli-Q-water, ethanol (EtOH), methanol (MetOH), and DMSO as solvents.

### 4.2 Emulsions

#### 4.2.1 Interfacial Tension

Prior to emulsion preparation, the interfacial tension between oil and aqueous dispersions containing stabilizing particles was measured by means of the pendant drop technique by using an Attention Theta optical tensiometer (Biolin Scientific, Finland). The image of the droplet was recorded with a black and white digital camera, and the interfacial tension was obtained by iterative fitting of the shape of the droplet with the Young–Laplace equation. The droplet was formed using a 0.718 mm (22 gauge) stainless steel needle. The following samples were studied: OO and OO with curcumin (OO-Cur); the measurements were conducted at the water interface, CNC interface (0.5 wt % dispersion), and CAS interface (0.5 wt % solution). Each analysis was performed in triplicate.

#### 4.2.2 Emulsification

The formation of emulsions started with the preparation of CAS solution (2 wt %) by stirring CAS powder in Milli-Q water at ambient temperature for 4 h. Similarly, a CNC aqueous dispersion (2 wt %) was prepared by stirring CNC powder for 4 h followed by ultrasound treatment with a UP400 St sonicator (Hielscher, Germany) operating at 60% output for three cycles, each with a duration of 1 min. The emulsion oil phase was prepared by dispersing curcumin (15 mg) in OO (20 g). Due to the low solubility of curcumin, the oil phase was stirred for 24 h, followed by ultrasound treatment (UP400 St sonicator, 60% output, three 1 min cycles). The undissolved fraction of curcumin was removed by centrifugation (5 min at 6000 rpm), and the concentration of curcumin dissolved in OO was determined by UV/vis spectrophotometry using external calibration at λ = 423 nm. The curcumin concentration in the oil was 0.38 mg/g. Compared to medium chain triglycerides, where curcumin is more soluble (2.02 mg/g), OO has longer acyl chains and lower polarity, resulting in lower curcumin solubility. Depending on the dissolution conditions, curcumin solubility in OO varies (from 80 μg/mL to 0.45 mg/g).^3,37,38^

Emulsions with a particle content (CAS + CNC) of 0.5 wt %, an o/w ratio of 20/80 (w/w), and an emulsion mass of 10 g were formulated. OO and OO with curcumin (OO-Cur) were each emulsified in an aqueous phase by sonication (UP400 St, Hielscher, Germany). The emulsification is conducted according to ref 18 using three emulsification routes: E1, E2, and E3. In brief, droplets of E1 emulsions were prepared by adding a mixture of 4 g of CAS solution and 4 g of CNC dispersion (each with a concentration of 0.5 wt %) to 2 g of oil phase in a single step, after which the system was sonicated for 1 min. Droplets of E2 emulsions were prepared from the primary emulsion formed by the sonication of 2 g of oil with 3 g of 0.5 wt % CAS for 1 min. Then 5 g of CNC dispersion (0.5 wt %) was added to the primary emulsion, followed by sonication for 20 s, providing thus emulsions with an o/w ratio of 20/80. Finally, for E3 emulsions, the order of CAS and CNC additions was reversed. A primary emulsion stabilized by CNC (2 g of oil + 3 g of 0.5 wt % CNC dispersion) was prepared by sonication (1 min); a CAS dispersion (5 g of 0.5 wt % CAS) was then added to the primary emulsion followed by sonication (20 s), which again resulted in a final emulsion containing 20 wt % oil and 0.5 wt % particles. The emulsion’s aqueous phase contained 5 mM NaCl, which facilitated emulsification.^39^ Correspondingly, emulsions with a cryoprotectant were prepared. The cryoprotectants, SUC, d-GLU, or d-MAN, were each added in an amount of 1 g to the aqueous phase of the emulsion prior to the sonication of oil with the aqueous phase containing the respective stabilizer. This ensured their uniform distribution during the emulsion formation.

### 4.3 Characterization of Emulsions

#### 4.3.1 Size and Distribution

The size and distribution of emulsion droplets were measured by laser diffraction using a Malvern MasterSizer 3000 (Malvern Instruments Ltd.; UK) at 25 °C. Emulsion droplet absorbance and refractive index were set to 0.001 and 1.421, respectively. Mean droplet size was reported as the volume mean diameter (*D*(4; 3)).

#### 4.3.2 Microstructure

The microstructure of emulsion droplets was visualized by using an Olympus Fluoview FV3000 confocal laser microscope (Olympus, Japan). For imaging, the emulsions were viewed with the transmitted light channel of CLSM under 400× magnification. Images were acquired and processed with Olympus FV31S software.

### 4.4 Preparation of Freeze-Dried Emulsions

Prior to freeze-drying, the emulsions were frozen at −20 and then at −80 °C. The samples were transferred to a lyophilizer (Alpha 2–4/LSC basic, Martin Christ Gefriertrocknungsanlagen GmbH, Germany) set to −80 °C, and the main drying phase was started at a pressure of 0.06 mbar for 30 h, followed by the final drying phase run at a pressure of 0.001 mbar for 14 h. The prefix FDE denotes freeze-dried emulsions.

### 4.5 Characterization of Freeze-Dried Emulsions

#### 4.5.1 Microstructure

The microstructure of the samples was observed using a Phenom Pro X SEM at an accelerating voltage of 10 kV without the samples being covered with a layer of gold.

#### 4.5.2 Curcumin Release from Freeze-Dried Emulsions

Curcumin release was followed using a sink medium containing PBS with 0.5 wt % Tween 80. A sample of freeze-dried emulsion (0.5 g) was placed into a dialysis membrane (MWCO 3.5 kDa), and the release of curcumin into the continuously stirred medium (10 mL) was observed for 72 h. To maintain a constant volume of the sink medium, 2 mL of the solution was withdrawn at predetermined time intervals and replaced with an equal volume of fresh medium. The amount of curcumin released into the medium was determined by measuring the absorbance by means of UV/vis spectrophotometry (V-750, Jasco, USA) at a wavelength of 424 nm. The cumulative release of curcumin was calculated using the equation % *R* = (*m*/*m*_c_) × 100 (where % *R* equals the amount of curcumin released, *m* is the mass of released curcumin (mg) in 1 mL, and *m*_c_ is the mass of curcumin in the sample prior to freeze-drying).

#### 4.5.3 Redispersion from Freeze-Dried Samples

The freeze-dried samples were transferred to Eppendorf vials, to which Milli-Q water was added until the mass of the FDE and water was 50/50; subsequently, the samples were vortexed for 30 s. In the following step, each of these concentrated emulsions was diluted with water to an o/w ratio of 20/80, which corresponded to the ratio in the original emulsions. The emulsions were then visually examined to determine whether the sample had redispersed to emulsion droplets and to check for the presence of agglomerates. The reconstituted samples were observed by optical microscopy on an Olympus CX41 optical microscope (Olympus Corp., Japan) with 100–400× magnification. The figures were captured and used to determine the mean sizes of the droplets. For image analysis, ImageJ software was used.

#### 4.5.4 Antioxidation Activity

The antioxidation activity of emulsions was determined by means of their free radical-scavenging activity against DPPH radicals.^31^ A freeze-dried sample (50 mg) containing 0.0345 mg of curcumin was mixed with 2 mL of ethanol and vortexed for 30 s. The clear yellow ethanolic curcumin solution (1 mL) was mixed with 0.1 M ethanolic DPPH (1 mL). To make a blank solution, ethanol (1 mL) was mixed with an ethanolic curcumin solution (1 mL). A reference sample without curcumin was prepared by mixing 1 mL of ethanol with 1 mL of a DPPH solution. All samples were kept in the dark, and radical scavenging activity was determined by recording sample absorbances at λ = 515.7 nm for 60 min using a UV/vis spectrophotometer. The antioxidation activity was calculated using the following equation: AA (%) = 1 – (*A*_s_ – *A*_b_/*A*_r_) × 100, where *A*_s_, *A*_b_, and *A*_r_ are the absorbances of the sample, blank, and reference solutions, respectively. The Dean-Dixon method was used to calculate the means and standard deviations.

### 4.6 Biological Performance

#### 4.6.1 Transdermal Penetration

The transdermal penetration of curcumin from freeze-dried emulsions was determined on an in vitro porcine ear model using Franz diffusion cells according to OECD guidelines^40^ with minor modifications. Fresh porcine ears were obtained from a local slaughterhouse. Transepidermal water loss was measured using the TewameterTM (Courage+Khazaka, Köln, Germany) to evaluate the integrity of the skin. Since transepidermal water loss was 9.6 ± 1.6 g/m^2^/h, the skin integrity was assessed as good. Pieces of the skin were mounted on the diffusion cells filled with receptor fluid consisting of PBS buffer with Brij 98 (1.5%) and gentamicin sulfate (0.05%), and the temperature of the water bath was set to 32 ± 1 °C. For the penetration test, each of the freeze-dried curcumin emulsions was mixed with water in a ratio of 1:1 and vortexed; then, 100 μL of the sample was applied onto the skin in the donor compartments of the cells. After 24 h of penetration, the quantity of curcumin in/on each of the following was determined: (1) the donor chamber (cap; rinsed with 1 mL of methanol); (2) the surface of the skin; (3) the *stratum corneum* (using six tape strips); (4) the e*pidermis* and *dermis*, which were mechanically separated; and (5) the receptor fluid. Curcumin was extracted from the tape strips and skin with 1 mL of methanol using the vortex (30 s) before measurement. After filtration of the samples with a 0.2 μm syringe filter, the curcumin content in the individual fractions was determined using UV/vis spectrophotometry (JASCO V-750) at a wavelength of 418 nm using an external calibration curve.

#### 4.6.2 Cytotoxicity

The cytotoxicity of curcumin emulsions was determined according to the protocol of the ISO standard 10993-5 with minor modifications. Prior to testing, samples were pretreated by immersing them in liquid nitrogen for 2 h. After this sterilization step, the emulsions were added to the mouse embryonic fibroblast cells at 50, 25, 10, 5, 2, and 1% dilutions of the initial emulsion, corresponding to 38, 19, 7.6, 3.8, 1.52, and 0.76 μg of applied curcumin. After 24 h, cell viability was determined by MTT assay. Cells were incubated at 37 °C in 5% CO_2_ in humidified air, and their viability was determined using MTT cell proliferation assay kit (Duchefa Biochemie, The Netherlands). Absorbance was measured at λ = 570 nm, and the reference wavelength was adjusted to 690 nm. The results are presented as the percentage reduction in cell viability compared to cells cultivated in a medium without the tested materials. All tests were run in quadruplicates.

### 4.7 Statistical Analysis

Experiments related to physicochemical characterization were performed at least in triplicates, and data from these are presented as mean ± standard deviations. Transdermal penetration was conducted in triplicate, and cytotoxicity values are based on the test conducted in quadruplicate. Means ± standard deviations are reported. Statistical analysis was performed using GraphPad Prism version 6.01 for Windows, GraphPad Software, La Jolla California USA. Statistical differences were tested by a one-sample *t*-test. *P* < 0.05 was taken to indicate significant differences between data mean values.
